# Late-phase strategies to overcome limitations of PD-1/PD-L1 therapy: a clinical development trajectory through 2030

**DOI:** 10.3389/fonc.2026.1875861

**Published:** 2026-07-20

**Authors:** Elena A. Andresyuk, Natalya O. Porozova, Georgii D. Londaridze, Ivan S. Moiseev, Polina V. Kotselyabina, Yuri B. Porozov

**Affiliations:** 1 The Laboratory of Bio- and Chemoinformatics, School of Computer Science, Physics and Technology, HSE University, St. Petersburg, Russia; 2 Department of AI, Medsi Clinic, Moscow, Russia; 3 Raisa Maximovna (RM) Gorbacheva Research Institute, Pavlov Medical University, St. Petersburg, Russia; 4 Advitam Laboratory, Belgrade, Serbia

**Keywords:** biomarkers, Combination strategies, Immune checkpoint blockade, late-phase development, PD-1, PD-L1, registry-based review, Resistance to immunotherapy

## Abstract

Inhibition of the PD-1/PD-L1 axis has become a clinical standard across multiple malignancies, yet durable benefit remains restricted to selected settings, and many biologically rational combinations fail to maintain clinical benefit in late-phase evaluation. This structured, registry-derived narrative review, supported by expert-guided interpretation, was designed to identify the most clinically meaningful development directions among strategies intended to overcome the limitations of PD-1/PD-L1 inhibitor therapy and most likely to generate practice-relevant late-phase readouts through 2030. We searched major international and regional clinical trial registries and applied harmonization, cross-registry deduplication, formal late-phase filtering, semi-automated prioritization, and manual documentation-based verification. The final analytic corpus was interpreted using a two-level framework: first, by the dominant clinicobiological barrier limiting PD-1/PD-L1 efficacy, and second, by three criteria of clinical promise - exposure and effect, manageable toxicity, and confirmable clinical benefit. This approach narrowed a registry-derived pool of more than 13, 000 PD-1/PD-L1 studies to 47 clinically relevant late-phase programs that define the most likely near-term development trajectory through 2030. Within this corpus, a narrower confirmatory subset emerged in which incremental clinical benefit is tested most directly in comparative designs. These programs represent the most plausible leading strategies of the next phase of PD-1/PD-L1-based clinical development and provide a clinically interpretable framework for prioritizing future translational and late-phase efforts.

## 1 Introduction

Inhibition of the programmed cell death protein 1/programmed death-ligand 1 (PD-1/PD-L1) axis has become part of the clinical standards of anticancer drug therapy over the past decade (1). Clinically meaningful and durable benefit, however, remains confined to selected patient subgroups and specific clinical settings, whereas variability in response is shaped by multiple factors, including tumor-intrinsic and microenvironmental features, patient functional status and comorbidities, as well as the context of prior treatment (2–4).

As evidence has accumulated and indications have expanded, the focus of development has shifted from demonstrating activity to defining the conditions under which benefit is confirmed in late-phase clinical development and achieved with an acceptable safety profile suitable for broader use (1, 3). PD-1/PD-L1 blockade is increasingly viewed as a backbone component of combination strategies with chemotherapy, radiotherapy, antiangiogenic agents, targeted therapies, and other approaches; however, the clinical value of such combinations varies substantially and is often constrained by toxicity and by the practical feasibility of the regimen (1, 3). This, in part, reflects the fact that tumors do not uniformly respond to PD-1/PD-L1 blockade because of recurrent clinicobiological mechanisms that limit therapeutic efficacy (2, 5). Early efficacy signals are frequently attenuated when scaled to broader populations, and the relationship between surrogate endpoints and long-term clinical outcomes requires careful interpretation (3, 6, 7). Accordingly, when assessing the near-term prospects of emerging strategies, it is important to consider not only which mechanism of limited efficacy a given strategy is designed to address, but also whether the anticipated clinical benefit is maintained when the strategy is tested in late-phase evaluation (3, 8).

This Review aims to identify and interpret clinically meaningful development directions for strategies intended to overcome the limitations of PD-1/PD-L1 inhibitor therapy and most likely to generate clinically important readouts in the foreseeable future. To this end, we analyzed the major mechanisms limiting efficacy, the strategies being developed to address them, and late-phase clinical programs expected to complete within the 2026–2030 window. The interpretive framework proposed here comprises two steps: assigning each clinical strategy to a biologically grounded barrier to efficacy and evaluating its alignment with three complementary criteria of clinical promise: exposure and effect, manageable toxicity, and confirmable clinical benefit. In this framework, biological rationale is treated as a necessary but not sufficient condition; introducing more stringent interpretive criteria and helping to distinguish the most promising core set of programs from the broader and increasingly complex PD-1/PD-L1 development landscape. Therefore, this Review should be understood as a structured, registry-derived narrative review with expert-guided interpretation, rather than as a full systematic review, meta-analysis, or predictive modeling study. Viewed through this lens, the field may be interpreted in terms of which disease settings and clinical scenarios are most likely to yield PD-1/PD-L1-based strategies capable of shaping clinical practice in the foreseeable future.

## 2 Methods

### 2.1 Registry-based screening, deduplication, and assembly of the final analytic corpus

**Figure 1:**
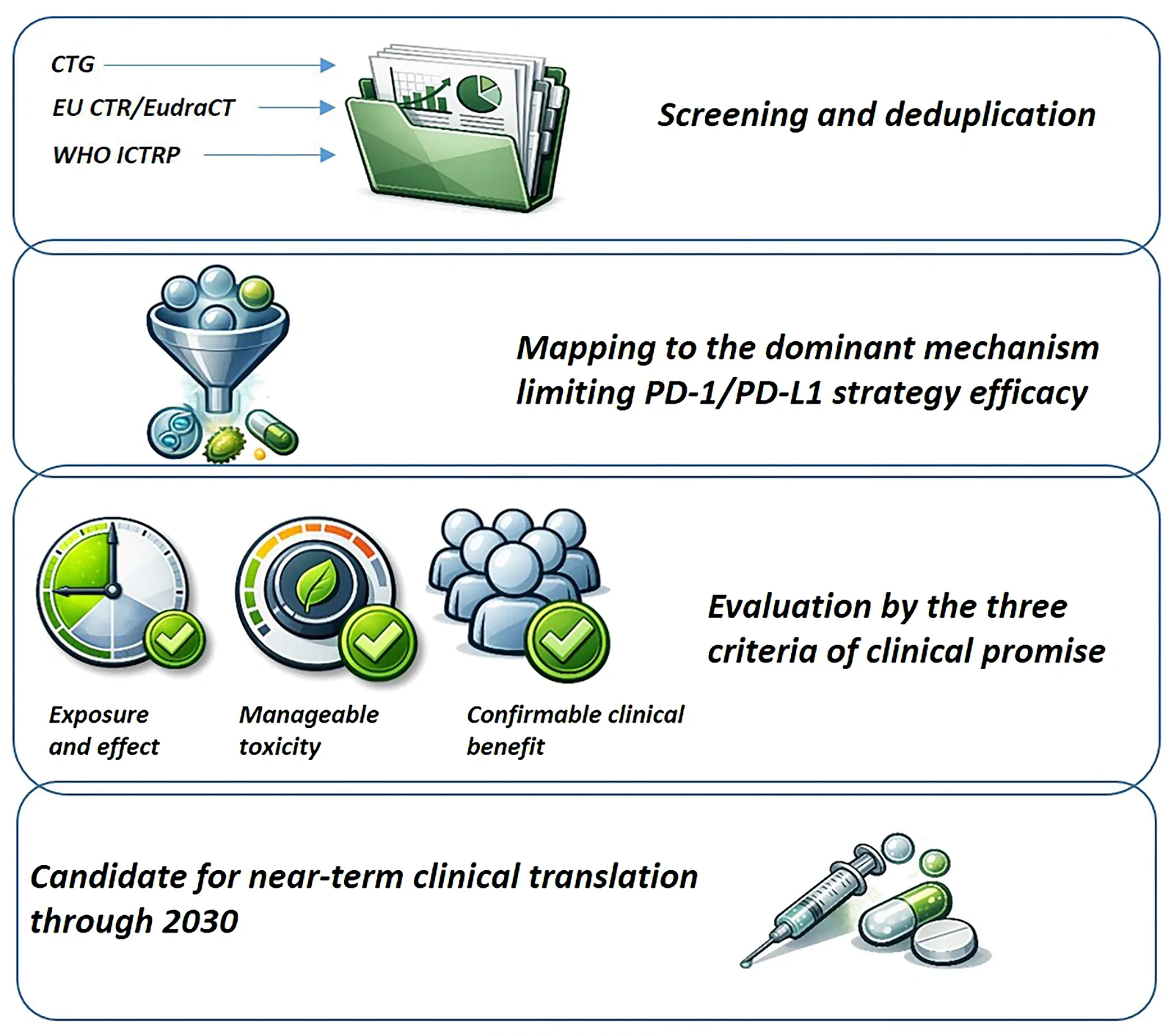
Study workflow from registry search to final analytic corpus.

**Figure 2:**
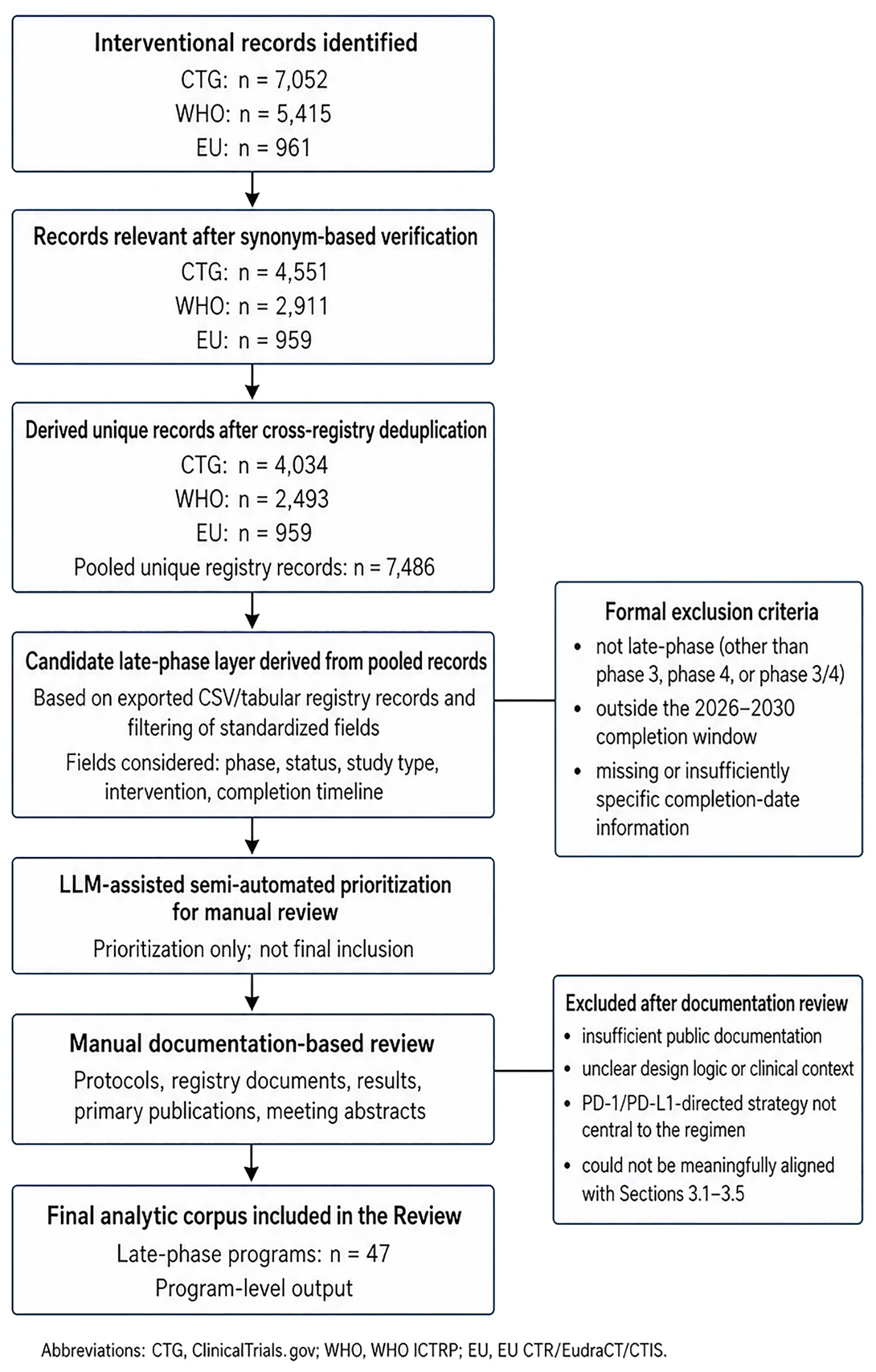
PRISMA-style flow diagram of registry screening and derivation of the candidate late-phase layer.

This Review used a registry-derived analysis of clinical trial records and incorporated selected elements of structured study selection. However, the workflow did not constitute a full systematic review, meta-analysis, or predictive modeling study. To address this aim, we assembled a final analytic corpus of late-phase programs most informative for identifying clinically meaningful leading directions in the development of PD-1/PD-L1-directed strategies through 2030. A fixed-snapshot approach was used together with selected PRISMA 2020 elements, without meta-analytic synthesis (9). The overall workflow is summarized in **Figure 1**, registry sources are listed in **Table 1**, and quantitative record flow is presented in **Figure 2**.

**Table 1:** Registry sources, coverage, and live search links used for data retrieval.

Registry	Coverage/scope	Live query link
ClinicalTrials.gov	Global (US and non-US entries)	https://www.clinicaltrials.gov/search?term=PD-1%20OR%20PD-L1
EU CTR/EudraCT (legacy)	EU/EEA interventional trials	https://www.clinicaltrialsregister.eu/ctr-search/search?query=PD-1+OR+PD-L1
WHO ICTRP	Aggregator of multiple registries	https://trialsearch.who.int/?query=PD-1+OR+PD-L1 (On the ICTRP results page, click Back to search and uncheck “COVID-19 trials only” (if selected), then re-run the search)

The table lists the registry sources used for record retrieval, together with their coverage and live search links. Because registry indexing is dynamic, the links may display updated results at the time of access. Snapshot date: October 10, 2025. For the European registry layer, legacy records under Directive 2001/20/EC were identified through EU CTR/EudraCT, whereas current public access under Regulation (EU) No 536/2014 is provided through the Clinical Trials Information System (CTIS) public portal. CTIS also includes transitioned former EudraCT trials. Because registry indexing and public availability are dynamic, links may display updated results at the time of access.

Record-level screening was conducted across ClinicalTrials.gov (CTG), the European registry layer represented by EU CTR/EudraCT and, where applicable, the public CTIS interface, and the WHO ICTRP search portal. The initial search used the keywords “PD-1” and “PD-L, ” restricted to interventional studies in the relevant registry fields. Retrieved records were expanded and assessed for relevance using a controlled synonym dictionary (Data Sheet. **Supplementary Table 1A**-**S1E**), including alternative spellings, international nonproprietary names, brand names, development codes, and related designations. A record was considered relevant if a PD-1/PD-L1-directed strategy could be identified in fields describing the intervention or treatment regimen. Preclinical records and case reports were excluded.

Data from all sources were harmonized into a unified structure that included identifier, link, title, study type, status, phase, indication, intervention or combination, and key dates. Duplicate records were identified using cross-identifiers (NCT number, EudraCT number, TrialID); when these were unavailable, potential matches were reviewed manually by title, sponsor, and intervention, and were not merged if uncertainty remained. Summary counts before and after synonym-based relevance verification and deduplication are shown in **Figure 2**.

Quality control of synonym expansion and deduplication was performed by two independent reviewers (E.A.A. and Y.B.P.) on a stratified random sample of 50 records per registry (n = 150 in total), drawn from the candidate late-phase subpool. Each record was assessed against the prespecified match definition for relevance to a PD-1/PD-L1-directed strategy and against duplication criteria; discrepancies were resolved by discussion with reference to the original registry record.

After deduplication, a pooled set of unique records was obtained (n = 7, 486). From this set, a candidate late-phase layer was derived from exported registry tables (CSV/tabular outputs) by filtering standardized fields, including phase, status, study type, intervention description, and completion timelines. In this Review, late-phase trials were defined as Phase III, Phase IV, and Phase III/IV studies. When reported, the study completion date had to fall within 2026-2030, inclusive. Full calendar-year coverage was used for 2026, without month-level stratification, to avoid tying the sample to the review update date and to minimize bias related to delays between study completion, registry updating, and publication. Records lacking a reported estimated completion date, or lacking sufficient specificity for strict assignment to the 2026–2030 interval, were excluded at this stage.

The next step was semi-automated record prioritization supported by a large language model (GPT-5.2 to GPT-5.5 Thinking; accessed through the OpenAI web interface). The model served as an auxiliary tool for flagging records for priority manual review and organizing candidate programs according to predefined fields, including formal late-phase status, expected completion window, intervention type, availability of supporting public documentation, and preliminary relevance to the aims of the Review. The outputs consisted of priority flags, brief rationale notes, and suggested grouping of candidate records; they were used to support workflow organization and did not determine record eligibility, final inclusion, mechanistic assignment, or clinical interpretation.

Final inclusion in the analytic corpus was determined only after manual review of publicly available documentation, including study protocols, structured registry documents, published interim or final results, primary publications, and, where necessary, authoritative meeting abstracts. Programs were retained only when the documentation was sufficient to verify the design logic, clinical context, and central role of the PD-1/PD-L1-directed strategy within the regimen under study. An additional requirement was meaningful alignment with one of the dominant clinicobiological barriers discussed in Sections 3.1-3.5. Accordingly, the final set analyzed in this Review should be understood not as a direct output of registry screening, but as a manually verified analytic corpus of programs.

Although screening, harmonization, and deduplication were performed across three registry sources, the final corpus proved to be predominantly NCT-representable. Programs are therefore cited in the main text primarily by their most stable and widely used public identifier, most often the NCT number. In this context, CTIS and WHO ICTRP served mainly as corroborating and deduplication layers rather than as major sources of unique final inclusions.

For interpretation, registry metadata were integrated with study protocols, published interim results, primary publications, and review articles indexed in PubMed and Web of Science over the period of active clinical development (2014-October 10, 2025), including broader reviews addressing current challenges in immune checkpoint inhibitor therapy and the associated biological constraints (5).

### 2.2 Two-level framework for data interpretation

The two-level interpretive framework was applied to the final analytic corpus assembled after semi-automated prioritization and subsequent manual, documentation-based verification. Thus, only those programs were taken forward for detailed analysis for which it was possible to determine reliably which dominant clinicobiological barrier the strategy was intended to address and whether the design could plausibly yield a clinically meaningful late-phase outcome. This is consistent with the aim of the Review, which was not to catalog the entire late-phase landscape, but to identify the most clinically meaningful leading directions in the field.

At the first level, programs were mapped to biologically grounded mechanisms limiting the efficacy of PD-1/PD-L1 inhibitor therapy (5) in Section 3. Only those programs whose design logic could be credibly interpreted as addressing one of the dominant clinicobiological barriers described in Sections 3.1–3.5 were retained for detailed discussion.

At the second level, strategies were evaluated against three complementary criteria of clinical promise: exposure and effect, manageable toxicity, and confirmable clinical benefit (Section 4) (7, 8, 10–12);. The assessment was qualitative and integrated registry metadata, including phase, status, completion timelines, interventions, and endpoints, with available efficacy and safety data from the sources listed in **Table 2**. At this stage, registry data and documentary sources were used not only to confirm the formal late-phase status of a program, but also to assess whether its design provided an interpretable test of the biological and clinical question relevant to the aims of the Review.

**Table 2:** Sources of information on clinical programs.

Level/criterion	Source of information by criterion (in order of priority)
Dominant mechanism limiting the efficacy of PD-1/PD-L1 inhibitor therapy	Registries (study design, comparator groups, and combination rationale): ClinicalTrials.gov, CTIS (EU), EMA overview of CTIS; publications/mechanistic rationale: PubMed; conference materials (if no full article is available): ESMO abstracts
Exposure and effect	Regulatory documents (when available): FDA Exposure–Response Guidance, FDA approvals & reviews, EMA EPARs; publications: PubMed; protocol and SAP in registry appendices: protocol/SAP PDF files appended to registry records; dose–response principle: ICH E4 (via EMA)
Manageable toxicity	Registries (structured results, AE/SAE): ClinicalTrials.gov results reporting, ClinicalTrials.gov results definitions; publications (AE details, discontinuations, reasons for treatment modification): PubMed; prescribing information/warnings and toxicity management (when approved): FDA labeling overview, EMA EPARs; clinical guidance on irAEs: ASCO guideline update (JCO), 10.1200/JCO.21.01440
Confirmable clinical benefit	Registries (phase, design, primary endpoint, sample size, completion timelines): ClinicalTrials.gov, CTIS (EU); late-phase publications and/or primary endpoint readouts: PubMed; regulatory decisions/assessments (when relevant): FDA approvals & reviews, EMA EPARs; publications on endpoint selection and interpretation of surrogate outcomes: TRIPOD statement, 10.7326/M14-0697

Strategies whose principal signal relied predominantly on surrogate endpoints without subsequent confirmation of clinically meaningful benefit were regarded as less convincing candidates for regulatory approval and clinical implementation (6, 8). Conversely, neutral or negative confirmatory results were interpreted as defining the limits of a strategy’s applicability, that is, situations in which an early signal was not sustained under more rigorous study design and broader clinical testing.

The structured fields used in this Review may provide a basis for future computational extensions of this approach, including the encoding of clinical programs as feature vectors, exploratory clustering, and multidimensional visualization (13). However, no quantitative embedding of clinical programs, computational clustering, or predictive modeling of the probability of confirmable clinical benefit was performed in the present work.

## 3 Major biological mechanisms limiting PD-1/PD-L1 efficacy and the rationale for combination strategies

Over the past decade, the clinical development of PD-1/PD-L1 inhibitors as backbone components of treatment regimens has expanded substantially. In routine clinical practice, PD-1/PD-L1 monotherapy has largely given way to combination regimens (3). In selected settings, however, monotherapy remains justified. For example, in the first-line treatment of non-small-cell lung cancer (NSCLC), pembrolizumab demonstrated an overall survival benefit versus platinum-based chemotherapy in patients with PD-L1-expressing disease, including the subgroup with high PD-L1 expression and no sensitizing EGFR or ALK alterations, in KEYNOTE-042 (14). However, this remains more the exception than the rule. The shift toward combination regimens has been driven by barriers to response and resistance, that is, recurrent biological mechanisms that can substantially limit the efficacy of PD-1/PD-L1 inhibitor monotherapy, such that even biologically well-rationalized combinations frequently yield neutral or negative results. In this section, clinical programs are organized according to the major biological mechanisms limiting PD-1/PD-L1 efficacy that most often shape development outcomes (5). These categories should be understood as dominant organizing axes rather than as mutually exclusive biological classes, because several programs may have overlapping mechanistic effects.

### 3.1 Weak initiation of the T-cell response and limited expansion of the T-cell repertoire

When baseline antitumor immune activity is low and antigen presentation is insufficient, several treatment modalities, including cytotoxic chemotherapy, radiotherapy, their combination as concurrent chemoradiotherapy (CRT), and personalized vaccines, can enhance tumor antigen release and proinflammatory signaling, including through immunogenic tumor cell death. This may create conditions that increase the likelihood of clinical response to PD-1/PD-L1 inhibitors (15, 16).

In metastatic NSCLC, pembrolizumab combined with pemetrexed and platinum significantly improved progression-free survival (PFS) and overall survival (OS) versus chemotherapy alone: in the primary 2018 analysis of KEYNOTE-189, median PFS was 8.8 months in the pembrolizumab plus pemetrexed-platinum group versus 4.9 months in the placebo plus pemetrexed-platinum group, and the risk of death was reduced by approximately 51% (17). Updated analyses were subsequently reported, including the 5-year outcomes published in 2023 (18). Similarly, in unresectable stage III NSCLC, durvalumab consolidation after concurrent chemoradiotherapy improved survival versus placebo in PACIFIC: median PFS was 16.8-16.9 versus 5.6 months, median OS was 47.5 versus 29.1 months, and the estimated 5-year OS rate was 42.9% versus 33.4%, respectively (19, 20).

Looking toward 2030, late-phase clinical programs combining chemotherapy and/or radiotherapy with PD-1/PD-L1 inhibitors are expected to clarify the clinical settings in which these regimens provide not only early disease control but also durable gains in clinically meaningful outcomes. One key example is KEYLYNK-012 (NCT04380636), in which pembrolizumab is administered with concurrent chemoradiotherapy and then continued with or without olaparib. The published study design supports this treatment schema (21), whereas the estimated completion date should be taken from the registry record rather than from the article itself.

In another disease setting, namely locally advanced unresectable esophageal cancer and gastroesophageal junction tumors, the phase III KEYNOTE-975 trial (NCT04210115) evaluates the addition of pembrolizumab to definitive chemoradiotherapy versus placebo given with the same chemoradiotherapy backbone; the dual primary endpoints are OS and event-free survival (EFS) (22). More examples are provided in **Table 3**. The reported primary completion date should be taken from the registry entry rather than inferred from the design paper.

**Table 3:** Examples of late-phase clinical programs grouped by the major clinicobiological mechanism limiting efficacy: weak initiation of the T-cell response and limited expansion of the T-cell repertoire.

Regimen	Program (NCT)	Indication/setting	Key late-phase milestone by 2030	References
PD-1 + concurrent chemoradiotherapy → maintenance phase (pembrolizumab)	KEYNOTE-A18/ENGOT-cx11/GOG-3047 (NCT04221945)	High-risk, locally advanced cervical cancer; primary treatment	26 Jan 2026	(23)
PD-1 + concurrent chemoradiotherapy → maintenance phase (pembrolizumab ± olaparib	KEYLYNK-013 (NCT04624204)	Limited-stage SCLC; primary treatment	28 Oct 2027	(24)
PD-L1-based consolidation after concurrent/sequential CRT (durvalumab)	PACIFIC-5 (NCT03706690)	Unresectable stage III NSCLC without progression after definitive platinum-based concurrent CRT	26 Feb 2027	(25)
PD-1 + chemotherapy → surgery → adjuvant phase (pembrolizumab)	KEYNOTE-671 (NCT03425643)	Resectable stage II-IIIB (N2) NSCLC; perioperative treatment	29 Jun 2026	(26, 27)
PD-1 before surgery → surgery → postoperative radiotherapy ± cisplatin + pembrolizumab → adjuvant phase (pembrolizumab	KEYNOTE-689 (NCT03765918)	Resectable locally advanced head and neck squamous cell carcinoma; perioperative treatment	10 Sep 2026	(28)

CRT, chemoradiotherapy.

INTerpath-001 (NCT05933577) also warrants separate mention, because the same mechanistic logic of enhancing antitumor response initiation and expanding the tumor-specific T-cell repertoire is implemented not through CRT, but through the addition of the personalized neoantigen vaccine V940 to adjuvant PD-1 blockade. Late-phase development of the same platform is ongoing in another disease setting: the phase III INTerpath-002 trial (NCT06077760) evaluates V940 plus pembrolizumab in the adjuvant setting in patients with completely resected stage II-IIIB NSCLC. Key late-phase readouts for INTerpath-001 are expected in October 2029 for the primary analysis, with full study completion anticipated in September 2030; for INTerpath-002, the corresponding dates are expected in June 2030 and December 2035.

Overall, for category 3.1, we identified nine late-phase clinical programs for which key late-phase milestones are expected by 2030.

### 3.2 Immune exclusion and angiogenesis-driven vascular-stromal barriers

The immune-excluded phenotype is characterized by the failure of T cells to penetrate the tumor or by their rapid loss of function within the tumor tissue, whereas VEGF-dependent vascular and stromal mechanisms sustain this barrier through endothelial anergy, impaired perfusion and hypoxia, and reduced recruitment of effector cells (29–32). Accordingly, VEGF/VEGFR inhibition is viewed as a means of partially alleviating the vascular-stromal barrier and widening the window for effective PD-1/PD-L1 blockade, thereby providing the biological rationale for PD-1/PD-L1×VEGF/VEGFR-directed programs, including bispecific antibodies (31, 32).

Ivonescimab (PD-1×VEGF-A) illustrates this class particularly well, from early signals of activity to testing the reproducibility of clinical benefit (33–35). Ivonescimab is being evaluated in several phase III NSCLC programs, including HARMONi-2 (NCT05499390), HARMONi-3 (NCT05899608), and HARMONi-6 (NCT05840016). For HARMONi-2 and HARMONi-6, key efficacy results have already been reported and include a PFS advantage over PD-1-directed comparators (34, 35). In HARMONi-2, median PFS was 11.1 months versus 5.8 months (HR 0.51) in favor of ivonescimab (34). In HARMONi-6, median PFS was 11.1 months versus 6.9 months (HR 0.60) in favor of ivonescimab plus chemotherapy (35).

At the same time, OS remains the key uncertainty. Even with convincing PFS shifts, the clinical strength of the class in broader populations will ultimately depend on mature OS updates and independent confirmation of the late-phase signal, a point explicitly emphasized for ivonescimab in a Lancet commentary (33). For HARMONi-3, the trajectory of near-term key late-phase milestones has already been outlined in company and SEC disclosures: screening for the squamous cohort was completed in the first quarter of 2026, an interim PFS analysis is expected in the second quarter of 2026 with OS still immature, the final PFS analysis for the squamous cohort is expected in the second half of 2026, and the final PFS analysis for the non-squamous cohort is expected in the first half of 2027. Thus, late-stage risk for this class may depend less on the absence of biological activity than on insufficient reproducibility and/or an insufficient magnitude of OS benefit (32, 33). Examples of programs grouped under this barrier are shown in **Table 4**.

**Table 4:** Examples of late-phase clinical programs grouped by the major biological mechanism limiting PD-1/PD-L1 efficacy: immune exclusion and angiogenesis-driven vascular-stromal barriers.

Regimen	Program (NCT)	Indication/setting	Key late-phase milestone by 2030	References
PD-1×VEGF bispecific antibody + chemotherapy	AK112-301/HARMONi-A (NCT05184712)	EGFR-mutant unresectable locally advanced or metastatic non-squamous NSCLC after EGFR-TKI	Sep 2026	(36)
PD-L1×VEGF bispecific antibody + chemotherapy	ROSETTA-Lung-01 (NCT06712355)	Extensive-stage SCLC, first-line treatment	Mar 2029	(37)
PD-L1×VEGF bispecific antibody + paclitaxel	PM8002 + paclitaxel (NCT06616532)	SCLC, second-line treatment	25 Dec 2028	(38)(prior phase II signal)
PD-1 + VEGFR multikinase inhibitor + TACE	LEAP-012 (NCT04246177)	Unresectable non-metastatic HCC treated with TACE	25 Mar 2026	(39)
PD-1 + VEGFR multikinase inhibitor + chemotherapy	LEAP-014 (NCT04949256)	Advanced/metastatic esophageal squamous cell carcinoma, first-line treatment	12 Jun 2026	(40)
PD-1 + VEGFR multikinase inhibitor + chemotherapy	LEAP-015 (NCT04662710)	HER2-negative gastroesophageal adenocarcinoma, first-line treatment	31 Mar 2026	(41)

Depending on study design and reporting structure, the milestone shown may correspond to primary analysis, primary completion, study completion, or another clinically meaningful confirmatory update expected within the 2026–2030 window.

EGFR-TKI, epidermal growth factor receptor tyrosine kinase inhibitor; HCC, hepatocellular carcinoma; TACE, transarterial chemoembolization.

Overall, manual verification of clinical trial registries identified 14 strictly relevant late-phase programs for this mechanism, with key late-phase milestones expected in 2026-2030; when regimens containing an additional antiangiogenic component were included more broadly, the pool increased to 17 programs (**Supplementary Table 2A**).

### 3.3 Myeloid-dominated immunosuppression and redundancy of coinhibitory pathways

Redundancy of coinhibitory pathways is one of the major mechanisms through which myeloid-dominated immunosuppression is established within the tumor microenvironment (42, 43). Suppressive myeloid cells not only constrain effector T-cell activity through cytokines, metabolic factors, and cell-cell interactions, but also sustain additional inhibitory signaling pathways, including the PD-L1, VISTA, TIGIT/CD155, and TIM-3/Gal-9 axes, which can maintain T-cell dysfunction after PD-1/PD-L1 blockade (42–44). Under these conditions, limited efficacy of monotherapy directed against a single immune checkpoint may indicate that immunosuppression is driven not by one isolated axis, but by several overlapping suppressive mechanisms sustained by the myeloid tumor microenvironment (45, 46). The addition of a second immunoregulatory target is therefore justified only when it addresses a complementary mechanism limiting efficacy rather than duplicating the effect on an already inhibited immune axis. Otherwise, combination therapy more often increases toxicity without yielding durable clinical gain (47–49). In SKYSCRAPER-01 (NCT04294810), the addition of tiragolumab (anti-TIGIT) to atezolizumab (anti-PD-L1) did not improve overall survival versus atezolizumab monotherapy, illustrating that intensification of coinhibitory blockade alone does not guarantee late-phase success even in PD-L1-high populations (50, 51).

In this context, programs based on conditional PD-L1×4-1BB costimulation, such as acasunlimab/GEN1046, and bispecific formats combining dual checkpoint blockade with CTLA-4, including PD-1×CTLA-4 cadonilimab/AK104 and PD-L1×CTLA-4 KN046, must demonstrate additional clinical benefit in late-phase development beyond the effect expected from simple additive checkpoint modulation, while retaining acceptable tolerability. A positive late-phase example in this broader class already exists; however, it primarily reflects the overcoming of coinhibitory redundancy rather than direct targeting of myeloid immunosuppression. In COMPASSION-15, cadonilimab (PD-1×CTLA-4) combined with capecitabine and oxaliplatin (XELOX) improved overall survival and progression-free survival in patients with HER2-negative advanced gastric or gastroesophageal junction adenocarcinoma (52–58).

Looking toward 2030, the active pool within this subsection is most clearly represented by TIGIT-containing strategies, including domvanalimab and rilvegostomig, and by bispecific PD-1×CTLA-4 blockade with cadonilimab. Examples of the most stringent programs are shown in **Table 5**. In the available sources, we did not identify late-phase studies with direct myeloid-targeted intervention and key late-phase milestones within the 2026–2030 window, although such approaches are present at earlier stages of clinical development. Most directly myeloid-oriented programs remain preclinical or in phase I, including TREM2-, LILRB-, and part of the VISTA-directed landscape; VISTA warrants separate mention as an immune checkpoint particularly closely linked to myeloid cells (43, 63). The more mature programs identified in this subsection instead primarily involve combining PD-1/PD-L1 blockade with other immunoregulatory targets, most notably TIGIT and CTLA-4 (43, 46, 64). Manual verification of open registries identified 14 late-phase entries for this mechanism with key late-phase milestones in 2026-2030; however, the main text of this subsection includes only eight active programs in which the coinhibitory component remains central to the late-phase hypothesis under evaluation. In addition to the most stringent examples, we also included ARTEMIDE-Gastric01 (NCT06764875), NCT06566755, and ARTEMIDE-HCC01 (NCT06921785) in the overall count. In ARTEMIDE-Gastric01, rilvegostomig is incorporated into a HER2-directed design with trastuzumab deruxtecan and a fluoropyrimidine; however, the coinhibitory component remains one of the key elements of the strategy under comparison (65). In NCT06566755, cadonilimab is combined with chemotherapy and bevacizumab in patients with RAS-mutant or right-sided metastatic MSS colorectal cancer, such that coinhibitory and anti-VEGF logic are implemented simultaneously (66). In ARTEMIDE-HCC01, rilvegostomig is being evaluated in combination with bevacizumab with or without tremelimumab as first-line treatment for unresectable hepatocellular carcinoma, likewise reflecting a multi-axis but still coinhibitory-oriented design (67).

**Table 5:** Examples of clinical programs designed to overcome coinhibitory redundancy.

Regimen/key molecule	Program (NCT)	Indication/setting	Key late-phase milestone by 2030	References
anti-TIGIT-containing regimen: domvanalimab + zimberelimab + chemotherapy	STAR-121 (NCT05502237)	Metastatic NSCLC without actionable genomic alterations; first-line treatment	Jan 2029	(59)
PD-1/TIGIT bispecific antibody: rilvegostomig + platinum-based chemotherapy	ARTEMIDE-Lung02 (NCT06692738)	Metastatic squamous NSCLC with PD-L1 expression; first-line treatment	8 Oct 2029	(60)
PD-1/TIGIT bispecific antibody: rilvegostomig + platinum-based chemotherapy	ARTEMIDE-Lung03 (NCT06627647)	Metastatic non-squamous NSCLC with PD-L1 expression; first-line treatment	25 Mar 2030	(61)
PD-1/TIGIT bispecific antibody: rilvegostomig	ARTEMIDE-Lung04 (NCT06868277)	Metastatic PD-L1-high NSCLC; first-line treatment	2 Dec 2030	(62)
Bispecific PD-1×CTLA-4 blockade: cadonilimab + induction chemotherapy + concurrent chemoradiotherapy	GEMSTONE (NCT05587374)	High-risk locoregionally advanced nasopharyngeal carcinoma; induction chemotherapy + concurrent chemoradiotherapy	1 Aug 2029	Registry source preferred; current DOI is indirect

CRT, chemoradiotherapy; NPC, nasopharyngeal carcinoma.

### 3.4 Tumor-intrinsic mechanisms of immune escape and limited efficacy driven by tumor-cell signaling pathways

In this section, tumor-cell signaling pathways refer to tumor-intrinsic oncogenic and adaptive mechanisms, including receptor, metabolic, and stress-adaptive axes, that simultaneously sustain an aggressive tumor phenotype and shape intratumoral mechanisms of immune escape and limited sensitivity to PD-1/PD-L1 blockade. In some settings, when the tumor is defined by a key oncogenic alteration such as BRAF V600, combinations of PD-1/PD-L1 inhibitors with BRAF/MEK inhibition have been developed as an attempt to combine the rapid tumor control achieved with BRAF/MEK-targeted therapy with the potentially greater durability of PD-1/PD-L1 blockade, thereby reducing the risk of early progression in biologically aggressive disease (68, 69). Clinical testing of this rationale has produced mixed results. In the randomized double-blind phase II KEYNOTE-022 part 3 study in first-line BRAF V600-mutant advanced melanoma, the addition of pembrolizumab to dabrafenib and trametinib increased PFS to 16.0 versus 10.3 months (HR 0.66), but the effect did not meet the prespecified threshold for statistical significance (70). By contrast, in the randomized double-blind placebo-controlled phase III IMspire150 trial, the addition of atezolizumab to vemurafenib and cobimetinib produced a statistically significant improvement in PFS, from 10.6 to 15.1 months (HR 0.78) (69).

Among studies for which key late-phase milestones are expected by 2030, the logic of target-immunotherapy combinations will be tested beyond the BRAF/MEK setting, including in NSCLC with KRAS G12C mutation. The programs that most directly reflect this logic are trials in which PD-1 blockade is paired with direct inhibition of an intratumoral driver cascade, above all KRAS G12C; these include the phase II/III KRYSTAL-7 program (NCT04613596), whose phase III portion compares adagrasib plus pembrolizumab versus pembrolizumab alone (71), and Krascendo 2 (NCT06793215) (72), which represent the most direct clinical tests of the target-immunotherapy hypothesis within this mechanistic class.

Alongside these programs, the same broader conceptual cluster also includes multifactorial tumor-cell-directed strategies in which PD-1 blockade remains an essential component, while tumor-cell targeting is implemented not only through a canonical oncogenic driver but also through adaptive or receptor pathways linked to tumor aggressiveness and immune escape. In this group, we included combinations involving HIF-2α inhibition in LITESPARK-022 (NCT05239728) (73); registry-based milestone source remains preferable for later readouts), a DDR/PARP component in RUBY Part 2 (NCT03981796) (74, 75), CLDN18.2-directed tumor-cell targeting in LUCERNA (NCT06901531) (76), and an EGFR/MET-directed receptor component in OrigAMI-5 (NCT07276399). Brief program characteristics are provided in **Table 6**.

**Table 6:** Examples of clinical programs designed to overcome tumor-intrinsic mechanisms of immune escape and aggressive phenotype driven by tumor-cell signaling pathways.

Regimen/key molecule	Program (NCT)	Indication/setting	Key late-phase milestone by 2030	References
KRAS G12C + PD-1: adagrasib + pembrolizumab	KRYSTAL-7 (NCT04613596)	Locally advanced unresectable or metastatic NSCLC with KRAS G12C and PD-L1 TPS ≥50%; first-line treatment	31 Oct 2029	(71)
KRAS G12C + PD-1: divarasib + pembrolizumab	Krascendo 2 (NCT06793215)	Previously untreated advanced or metastatic non-squamous NSCLC with KRAS G12C; first-line treatment	31 Oct 2030	(72)
HIF-2α + PD-1: belzutifan + pembrolizumab	LITESPARK-022 (NCT05239728)	Clear cell renal cell carcinoma after nephrectomy, at increased risk of recurrence; adjuvant treatment	28 Sep 2029	(73, 77)
DDR/PARP + PD-1 + chemotherapy: dostarlimab + carboplatin/paclitaxel → dostarlimab + niraparib	RUBY Part 2 (NCT03981796)	Primary advanced or recurrent endometrial cancer; first-line treatment	26 Nov 2026	(78, 79)
CLDN18.2 + PD-1 + chemotherapy: zolbetuximab + pembrolizumab + chemotherapy	LUCERNA (NCT06901531)	HER2-negative, CLDN18.2-positive, PD-L1-positive locally advanced unresectable or metastatic gastric/gastroesophageal junction adenocarcinoma; first-line treatment	30 Sep 2028	(76)
EGFR/MET-directed regimen + PD-1 + carboplatin: amivantamab + pembrolizumab + carboplatin	OrigAMI-5 (NCT07276399)	HPV-unrelated recurrent/metastatic head and neck squamous cell carcinoma; first-line treatment	18 Jun 2029	Registry source

Depending on study design and reporting structure, the milestone shown may correspond to primary analysis, primary completion, study completion, or another clinically meaningful confirmatory update expected within the 2026–2030 window.

DDR, DNA damage response; HIF-2α, hypoxia-inducible factor 2 alpha; PARP, poly(ADP-ribose) polymerase.

It is also useful to distinguish studies that extend the same target-immunotherapy logic but are expected to yield primarily primary-analysis readouts within the time horizon considered here, whereas full protocol completion is expected later. This group includes, in particular, KRYSTAL-4 (NCT06875310) (62, 80) and KANDLELIT-007 (NCT07190248) (81).

Verification of open clinical trial registries identified 10 relevant late-phase programs for which key late-phase milestones are expected by 2030 and that align with the logic of tumor-intrinsic mechanisms limiting efficacy driven by tumor-cell signaling pathways. This set of studies comprises two main clusters: direct target-immunotherapy combinations for tumors with oncogenic driver alterations, most notably KRAS G12C, and multifactorial tumor-cell-directed strategies in which PD-1/PD-L1 blockade is combined with interventions targeting adaptive, receptor, or genome-instability-related pathways, including HIF-2α inhibition. Details are provided in **Supplementary Table 2B**. KEYLYNK-012/013 and LEAP-014/015 should also be noted as cross-relevant target-immunotherapy regimens, although they are discussed in detail in Sections 3.1 and 3.2.

### 3.5 Resensitization after failure of PD-1/PD-L1 inhibitor therapy

Clinical settings after failure of PD-1/PD-L1 inhibitor therapy represent the most stringent test for any combination strategy. In this context, transient immune activation or an isolated signal of activity is not sufficient; what is required is restoration of clinically meaningful disease control after progression on PD-1/PD-L1 blockade, together with confirmation that this effect is durable in late-phase evaluation. This is precisely why many regimens with promising early-phase results fail to reproduce their activity in more rigorous studies (2, 16).

No positive late-phase program convincingly demonstrating restoration of sensitivity after failure of PD-1/PD-L1 inhibitor therapy, and already capable of informing clinical practice, was identified in the open sources. On the contrary, completed rigorous evaluations underscore the difficulty of this challenge. In the phase III ILLUMINATE-301 trial (NCT03445533), the addition of tilsotolimod to ipilimumab in patients with advanced melanoma refractory to PD-1 inhibitor therapy improved neither objective response rate nor overall survival (82). Likewise, in the phase III PRAGMATICA-LUNG study (NCT05633602), ramucirumab plus pembrolizumab in previously treated patients with stage IV or recurrent NSCLC did not improve overall survival versus standard therapy (83).

At present, the feasibility of this broader approach is suggested mainly by earlier signals, including the randomized phase II SWOG S1616 study (NCT03033576) in metastatic melanoma refractory to PD-1/PD-L1 blockade (84), and the study Nivolumab With Ruxolitinib in Relapsed or Refractory Classical Hodgkin Lymphoma (NCT03681561), in which nivolumab plus ruxolitinib was evaluated in a phase I/II setting in relapsed or refractory classical Hodgkin lymphoma after prior immune checkpoint blockade (85).

Over the near-term horizon, IGNYTE-3 (NCT06264180, phase III) occupies a central position. In this study, a resistance-overcoming strategy continues to be tested in patients with advanced melanoma after anti-PD-1 and anti-CTLA-4 therapy; overall survival has been selected as the primary endpoint, and the key late-phase milestone falls within the time window of this Review (86); NCT06264180). Closely related late-phase programs include MarsLight-11 (NCT07217301, phase III) and PRESERVE-003 (NCT05671510, phase III). Both enroll patients with NSCLC after prior therapy containing PD-1/PD-L1 blockade and reflect attempts to overcome post-immunotherapy resistance in a late-phase setting; however, their logic extends beyond classical restoration of sensitivity to conventional PD-1/PD-L1 blockade, because the former uses the PD-1/IL-2 bispecific construct IBI363, whereas the latter evaluates the next-generation CTLA-4 antibody gotistobart (ONC-392/BNT316) against docetaxel. Stated as strictly as possible according to our methods, these programs constitute the current late-phase pool for the direction focused on restoration of sensitivity after failure of PD-1/PD-L1 inhibitor therapy.

## 4 Practical questions in late-phase clinical evaluation

Transition to phase III or another registration-enabling stage does not, in itself, guarantee either confirmed clinical benefit (87) or its reproduction on clinically meaningful endpoints (8) (88);. Section 3 presented examples of clinical studies with a well-substantiated biological rationale but neutral or negative late-phase outcomes. In this section, the selected directions are considered through the lens of three practical domains of late-phase clinical evaluation. This synthetic authorial framework is grounded in published principles of dose optimization (10, 11, 89), assessment of clinical benefit (87), and evaluation of toxicity in contemporary anticancer drug development (12, 90, 91), and comprises:

- Exposure and effect – the ability of a strategy to achieve exposure sufficient to produce an antitumor effect at a clinically usable dose and schedule; in contemporary oncology drug development, optimal dose selection is increasingly viewed not as a search for the maximum tolerated dose, but as an effort to align exposure, activity, and safety in a manner suitable for subsequent registration-enabling studies (10, 11, 89).
- Manageable toxicity – the ability to maintain an acceptable and predictable adverse-event profile within a dosing regimen suitable for clinical use, because toxicity often limits treatment duration, planned dose intensity, and the broader applicability of a combination in routine practice (12, 90, 91).
- Confirmable clinical benefit – the degree of confidence that the observed effect is reproducible on confirmatory endpoints. Publications addressing endpoint selection in immuno-oncology and interpretation of surrogate measures emphasize that early activity signals and surrogate outcomes are not equivalent to confirmed clinical benefit (7, 8, 92).

### 4.1 Biomarkers for patient selection and interpretation of PD-1/PD-L1-based strategies

Biomarkers may complement the clinical interpretation of therapeutic strategies at stages of development when confirmatory data from mature endpoints remain limited (see **Supplementary Table 3** for selected examples and assay-specific PD-L1 IHC considerations). In this role, they do not substitute for confirmation of clinical benefit on clinically meaningful outcomes, but rather reduce uncertainty in the interpretation of interim results and in patient selection, response interpretation, and trial design, while helping align the biological rationale of a strategy with already observable signals of activity (8, 93, 94). From a practical clinical perspective, these biomarkers should therefore be interpreted as complementary, context-dependent tools rather than as interchangeable predictors of benefit.

In the context of PD-1/PD-L1 blockade, among the most clinically validated markers are PD-L1 expression, which has been used for patient selection and is associated with a greater likelihood of benefit from first-line pembrolizumab in NSCLC (95), although its interpretation is limited by assay-specific scoring systems, spatial and temporal heterogeneity, and differences between tumor-cell and immune-cell staining (93, 95). MSI-H/dMMR status represents a biologically coherent predictor of sensitivity to PD-1 blockade, particularly in colorectal and other solid tumors, because it captures a hypermutated and immunogenic tumor state rather than a single ligand-expression readout (96, 97). In parallel, TMB may be considered a context-dependent indicator of tumor immunogenicity and neoantigen-generation probability, although its interpretation remains platform- and method-dependent and is influenced by threshold and tumor type, requiring further harmonization across assay systems (98, 99). For interim assessment of response depth and residual risk, ctDNA/MRD kinetics are attracting increasing interest, particularly in perioperative and resistance-overcoming settings, as they may further stratify recurrence risk in perioperative regimens that include an immunotherapy component and serve as an emerging tool for early biological and clinical verification of a strategy through dynamic assessment of residual disease, early molecular response, and recurrence risk rather than as a primary eligibility biomarker (100–102). Thus, in late-phase PD-1/PD-L1 development, biomarkers are most informative when they reduce uncertainty in patient selection, response interpretation, and trial design, whereas confirmation of clinical benefit must still rely on mature clinical endpoints (8, 94).

### 4.2 Clinically meaningful gain in antitumor activity

Any strategy aimed at improving the efficacy of PD-1/PD-L1 inhibitors ultimately seeks to achieve a clinically meaningful enhancement of antitumor effect. This, however, is achieved not only through pharmacologic optimization of exposure, but also through changes in the biological configuration of the treatment regimen. In solid tumors, this domain is particularly important because delivery and intratumoral distribution of antibodies and other large protein constructs are constrained by the properties of the tumor tissue, stroma, microenvironment, and target accessibility (103, 104). Historically, attempts have been made, and continue to be made, to enhance efficacy by intensifying modulation of an already selected immune axis. For PD-1/PD-L1 antibodies, however, this rarely becomes the dominant strategy, because within the clinically used dose range the gain in effect with increasing exposure is relatively limited. For pembrolizumab and nivolumab, for example, the available data do not support the view that increasing exposure within the clinically relevant range yields a clinically meaningful increase in effect (105–108). Likewise, in patients with metastatic NSCLC and PD-L1 TPS ≥50%, the addition of ipilimumab to pembrolizumab in KEYNOTE-598 improved neither OS nor PFS and was associated with greater toxicity (109).

Within the current late-phase pool, this logic is represented not by a single program, but by a series of late-phase studies with a PD-1×VEGF bispecific. In HARMONi-3 (NCT05899608), ivonescimab is being evaluated in combination with chemotherapy versus pembrolizumab plus chemotherapy in the first-line treatment of metastatic NSCLC, whereas in HARMONi-7 (NCT06767514) the same bispecific is being compared directly with pembrolizumab in patients with metastatic NSCLC and high PD-L1 expression. In this setting, the anticipated gain in effect is therefore viewed not as a consequence of simple intensification of PD-1 blockade, but as the result of simultaneously addressing vascular-stromal and immunosuppressive constraints within the tumor microenvironment (103, 110). For this class, a late-phase clinical signal is already available: in HARMONi-2, ivonescimab outperformed pembrolizumab in PFS in patients with PD-L1-positive advanced NSCLC, supporting the hypothesis that dual PD-1 and VEGF blockade may confer clinical benefit (34).

In INTerpath-001 (NCT05933577), late-phase testing has been extended to personalized neoantigen vaccination: V940 is added to adjuvant PD-1 blockade with the aim of enhancing antitumor effect through expansion of the tumor-specific T-cell repertoire; the study has a randomized phase III design (111). In PACIFIC-9, oleclumab or monalizumab is added to durvalumab consolidation after concurrent chemoradiotherapy, that is, a second immune component intended to alleviate CD73- or NKG2A-mediated immunosuppressive constraints in the microenvironment; this is likewise a randomized phase III study (112). Across these examples, the same design domain remains central: whether adding a second biologically rational mechanism can produce a clinically meaningful enhancement of antitumor effect sufficient to justify a more complex treatment regimen.

### 4.3 Manageable toxicity

A practical limitation of PD-1/PD-L1 inhibitor-based combination strategies remains immune-related adverse events (irAEs) or, more broadly, treatment-emergent adverse events (TEAEs), because these events often shorten the actual duration of therapy, increase the frequency of treatment interruptions and discontinuations, and may thereby diminish the anticipated clinical benefit. Aggregate evidence shows that, as treatment moves from monotherapy to more intensive immune combinations, both the frequency and severity of such events increase accordingly; in CheckMate 067, the incidence of grade 3–4 treatment-related adverse events was substantially higher with nivolumab plus ipilimumab than with nivolumab alone (113, 114).

Manageable toxicity therefore becomes a necessary condition for the clinical usability of a regimen. This logic is most clearly embedded in ABBIL1TY NSCLC-06 (NCT06635824), in which acasunlimab plus pembrolizumab is compared with docetaxel after prior PD-1/PD-L1-directed therapy and platinum-based chemotherapy; the engineering concept behind this construct is to make 4-1BB costimulation dependent on PD-L1 binding, thereby enhancing antitumor activity while maintaining tighter control of systemic immune activation (115, 116). A related question, implemented through a different engineering logic, is being tested in STAR-121 (NCT05502237), where domvanalimab and zimberelimab are added to chemotherapy in direct comparison with pembrolizumab plus chemotherapy, making safety and tolerability part of the late-phase evaluation alongside progression-free and overall survival (59). PRESERVE-003 (NCT05671510) further illustrates that, for next-generation anti-CTLA-4 strategies, manageable toxicity becomes a central component of the late-phase hypothesis: gotistobart is being compared with docetaxel in patients with metastatic squamous NSCLC after progression on PD-1/PD-L1 inhibitor therapy, and the program is explicitly designed to evaluate efficacy together with the safety profile (59). In GEMSTONE (NCT05587374), the issue of manageable toxicity arises at the level of the regimen itself. Cadonilimab is incorporated into an inherently intensive schedule of induction chemotherapy followed by concurrent chemoradiotherapy; thus, late-phase evaluation concerns not only efficacy, but also whether intensification of the immune component allows planned treatment to be delivered in full.

Accordingly, within our pool, the issue is not simply the search for less toxic regimens as such, but rather whether a new mechanism can be added without loss of manageable toxicity and without systematic erosion of planned treatment intensity.

### 4.4 Confirmable clinical benefit

The transition from an early signal to confirmed incremental clinical benefit remains one of the pivotal steps in the clinical selection of strategies on the path to regulatory approval. According to the report Clinical Development Success Rates and Contributing Factors (BIO/QLS/Informa), based on 12, 728 phase transitions across 9, 704 programs over 2011-2020, in oncology overall, 24.6% of programs entering phase II advance to phase III, and 43.9% of those that reach phase III ultimately achieve approval; for solid tumors, the corresponding figures are 23.4% and 39.8%, respectively. In other words, even after transition to phase III, only about two-fifths of oncology programs ultimately reach approval, and the proportion is even lower for solid tumors.

As specified in the Methods, the analysis included programs for which key late-phase milestones are expected by 2030, including primary readouts and subsequent clinically meaningful updates. Taken together, this late-phase set outlines the likely directions of further development in PD-1/PD-L1-based immunotherapy through 2030. A narrower subset within this Review is characterized by the most direct testing of incremental clinical benefit and therefore highlights the strategies closest to confirmation of clinical benefit and regulatory relevance. One such program is INTerpath-001, in which the contribution of a personalized neoantigen vaccine is tested in a direct add-on design, with a new component added to standard therapy (111). In PACIFIC-9, oleclumab or monalizumab is added to durvalumab consolidation after concurrent chemoradiotherapy, and these regimens are compared against durvalumab plus placebo; accordingly, the study addresses in equally direct fashion whether a second immune component provides additional clinical benefit when added to an established standard of care (112).

A different, but no less stringent, form of confirmation is represented by programs directly comparing a new regimen against the current standard. In HARMONi-3, ivonescimab plus chemotherapy is compared directly with pembrolizumab plus chemotherapy; in HARMONi-7, ivonescimab is compared with pembrolizumab in patients with metastatic NSCLC and high PD-L1 expression; in STAR-121, domvanalimab plus zimberelimab with chemotherapy is compared with pembrolizumab plus chemotherapy; and in ARTEMIDE-Lung04, rilvegostomig is compared directly with pembrolizumab in patients with metastatic PD-L1-high NSCLC. In all of these settings, the goal is not merely to demonstrate activity of a new regimen, but to establish a clinically meaningful gain over the current first-line pembrolizumab-based standard (59, 117–119).

ABBIL1TY NSCLC-06 (acasunlimab plus pembrolizumab) and PRESERVE-003 (gotistobart) also belong to this confirmatory subset, but they reflect a somewhat different clinical scenario: in these trials, new immune regimens are compared with a docetaxel-based control regimen in patients with progression after prior PD-1/PD-L1 inhibitor therapy and platinum-based chemotherapy, that is, in a more clinically challenging, pretreated, and refractory population (120, 121). Across these studies, the question of additional clinical benefit from a new component or a new regimen is posed with a minimal number of intermediate assumptions. Where available, biomarker-defined populations or longitudinal biomarker readouts may provide additional support for the interpretation of observed clinical benefit, but do not substitute for confirmation on mature endpoints.

## 5 Conclusion

In this Review, a structured two-step interpretive framework was applied to phase III and later-stage strategies centered on PD-1/PD-L1 inhibitor-based immunotherapy, with expected completion or key data readouts in the 2026–2030 window. This approach appropriately narrowed the overall registry-derived pool from more than 13, 000 records to a final set of 47 clinical PD-1/PD-L1 programs meeting the criteria of the present work. This compact final set reflects the stringency of the selection process and provides a clinically interpretable overview of the likely near-term development trajectory of the field through 2030. Within it, a narrower confirmatory subset can be distinguished, comprising programs in which clinical benefit is tested most directly in comparative designs; these are the studies we regard as the most promising strategies for the coming years. This does not imply that the remaining directions are weaker or less important. For a substantial proportion of late-phase programs, the dominant issues remain magnitude of effect, manageable toxicity, clinical usability of the regimen, and reproducibility of the signal in more complex populations.

Beyond this immediate interpretive function, the structured corpus assembled in this Review may provide a basis for future computational extensions of the proposed approach. Because each program can be described by categorical and semi-structured features, including mechanism class, indication, intervention type, endpoint design, toxicity profile, and completion timeline, the late-phase landscape could be encoded in future work to support exploratory clustering, multidimensional visualization using dimensionality-reduction methods such as t-SNE and UMAP, or model-assisted hypothesis generation. Such analyses may help determine whether recurrent combinations of mechanism, exposure, toxicity, and endpoint design are associated with confirmable clinical benefit in larger and prospectively updated datasets. In this sense, the present Review does not end with a static list of late-phase programs, but establishes a reproducible structure that can be updated, expanded, and quantitatively interrogated as the PD-1/PD-L1-directed therapy landscape evolves.

The practical value of this approach used in this study lies in its ability to move the 2030 outlook from the level of general expectations to that of clinically interpretable scenarios. What emerges is no longer an open-ended landscape of potentially active programs, but a set of concrete reference points. For clinicians, this provides a way to identify the programs most likely to influence treatment choices within the near-term horizon. For drug developers, it offers a basis for more precise prioritization of development directions in which late-phase risk has already been reduced, at least in part, by preceding clinical logic. For researchers and trial designers, it provides a tool for distinguishing strategies that have already entered a stage of stringent testing for incremental clinical benefit from those for which key questions of effect, manageable toxicity and feasibility remain unresolved.
