# Supplementary material for: Late-phase strategies to overcome limitations of PD-1/PD-L1 therapy: a clinical development trajectory through 2030
Source: Front Oncol. 2026 Jul 20;16:1875861. doi: 10.3389/fonc.2026.1875861 (PMC13454932; doi:10.3389/fonc.2026.1875861)
Supplement: Supplementary file 1 [file Table1.docx]

# **Data Sheet. Synonym Dictionary for PD-1/PD-L1**

Table S1A. PD-1/PD-L1 targeted monoclonal antibodies

| Target | INN | Brand | Development codes / synonyms | Sponsor | Region / status | First mention (year) | Reference |
| --- | --- | --- | --- | --- | --- | --- | --- |
| PD-1 | Balstilimab | — | AGEN2034 | Agenus | Development (late) | 2017 | (1) |
| PD-1 | Budigalimab | — | ABBV-181 | AbbVie | Development (late) | 2018 | (2) |
| PD-1 | Camrelizumab | AiRuiKa | SHR-1210 | Hengrui | CN approved | 2016 | (3) |
| PD-1 | Cemiplimab | Libtayo | REGN2810; SAR439684 | Regeneron/Sanofi | US/EU approved | 2016 | (4) |
| PD-1 | Dostarlimab | Jemperli | TSR-042; GSK-4057190 | GSK | US/EU approved | 2017 | (5) |
| PD-1 | Geptanolimab | Aibining | GB226 | Genor Biopharma | CN (r/r PTCL) | 2019 | (6) |
| PD-1 | MEDI0680 | — | MEDI0680; AMP-514 | AstraZeneca/MedImmune | Historical Phase 1/2 | 2014 | (7) |
| PD-1 | Nivolumab | Opdivo | BMS-936558; MDX-1106; ONO-4538 | BMS/ONO | US/EU/JP approved | 2010 | (8) |
| PD-1 | Pembrolizumab | Keytruda | MK-3475; SCH-900475 | Merck | US/EU/JP approved | 2012 | (9) |
| PD-1 | Penpulimab | Anike (安尼可) | AK105 | Akeso | CN/US updates | 2019 | (10) |
| PD-1 | Pidilizumab | — | CT-011 | CureTech/Medivation | Historical (target debated) | 2010 | (11) |
| PD-1 | Prolgolimab | — | BCD-100 | BIOCAD | RU (melanoma) | 2018 | (12) |
| PD-1 | Pucotenlimab | — | HX008 | Lepu Biopharma | CN (MSI-H; melanoma) | 2018 | (13) |
| PD-1 | Retifanlimab | Zynyz | INCMGA00012; MGA-012 | Incyte | US (MCC) approved | 2018 | (14) |
| PD-1 | Serplulimab | Hansizhuang | HLX10 | Henlius | CN labels | 2019 | (15) |
| PD-1 | Sintilimab | Tyvyt | IBI-308 | Innovent/Eli Lilly | CN approved | 2016 | (16) |
| PD-1 | Spartalizumab | — | PDR001 | Novartis | Development | 2016 | (17) |
| PD-1 | CS1003 | — | CS1003 | CStone | APAC Development | 2018 | (18) |
| PD-1 | Tislelizumab | Tevimbra | BGB-A317 | BeiGene | CN multi; US (ESCC) | 2017 | (19) |
| PD-1 | Toripalimab | Tuoyi / Loqtorzi | JS001 | Junshi/Coherus | CN multi; US (NPC) | 2016 | (20) |
| PD-1 | Zimberelimab | — | AB122; GLS-010 | Gloria/Arcus | CN (cHL; cervical) | 2018 | (21) |
| PD-L1 | Adebrelimab | Arelili / Ariely | SHR-1316; HTI-1088 | Hengrui/Shanghai Shengdi | CN approved | 2019 | (22) |
| PD-L1 | Atezolizumab | Tecentriq | MPDL3280A; RG7446 | Roche/Genentech | US/EU/JP approved | 2014 | (23) |
| PD-L1 | Avelumab | Bavencio | MSB0010718C | Merck KGaA/Pfizer | US/EU approved | 2015 | (24) |
| PD-L1 | BGB-A333 | — | BGB-A333 | BeiGene | Development | 2017 | (25) |
| PD-L1 | BMS-936559 | — | BMS-936559 | BMS | Early clinical (2012) | 2012 | (26) |
| PD-L1 | Cosibelimab | Unloxcyt | CK-301 | Checkpoint Therapeutics | US (CSCC) approved | 2018 | (27) |
| PD-L1 | CX-072 | — | CX-072; pacmilimab; CX-072 (Probody PD-L1) | CytomX | Phase 1/2 | 2017 | (28) |
| PD-L1 | Durvalumab | Imfinzi | MEDI4736 | AstraZeneca | US/EU/JP approved | 2014 | (29) |
| PD-L1 | Envafolimab | — | KN035 (subcutaneous) | Alphamab/3DMed/Simcere | CN approved | 2015 | (30) |
| PD-L1 | LY3300054 | — | LY3300054 | Eli Lilly | Development (early) | 2016 | (31) |
| PD-L1 | Sugemalimab | Cejemly | CS1001; GSK4086404 | CStone/GSK | CN approved | 2019 | (32) |
| PD-L1 | TQB2450 | — | TQB2450 | Chia Tai Tianqing | Development (CN) | 2020 | (33) |

Table S1B. Clinical small molecules targeting PD-1/PD-L1

| Target | Name | Development codes / synonyms | Mechanism | Clinical status | Sponsor | First mention (year) | Reference |
| --- | --- | --- | --- | --- | --- | --- | --- |
| PD-L1 | ABSK043 | ABSK043 | PD-L1 binder (oral) | FIH → Phase 2 (CN) | Abbisko | 2023 | (34) |
| PD-L1 | AN4005 | AN4005 | PD-L1 binder (oral) | Phase 1 | Adlai Nortye | 2024 | (35) |
| PD-L1 | CCX559 | CCX559 | PD-L1 binder (oral) | Phase 1 / company updates | ChemoCentryx/Amgen | 2023 | (36) |
| PD-L1 | Evixapodlin | GS-4224 | PD-L1 binder/dimerizer (oral) | Phase 1 | Gilead | 2024 | (37) |
| PD-L1 | INCB086550 | INCB086550 | PD-L1 binder/dimerizer (oral) | Early clinical (FIH) | Incyte | 2022 | (38) |
| PD-L1 | INCB099280 | INCB099280 | PD-L1 binder/dimerizer (oral) | Phase 1 | Incyte | 2024 | (39) |
| PD-L1 | MAX-10181 | MAX-10181 | PD-L1 binder/dimerizer (oral) | Phase 1 | MaxiNovel | 2019 | NCT04122339 |
| PD-L1 | YPD-30 (→ YPD-29B/IMMH-010) | IMMH-010; YPD-29B | PD-L1 binder/dimerizer (oral prodrug) | Phase 1 (CN) | CAMS/Partners | 2022 | (40) |
| PD-L1/VISTA | CA-170 | CA-170 | Dual PD-L1/VISTA modulator (oral) | Early trials; binding debated in the literature | Curis/Aurigene | 2017 | (41) |

Table S1C. PD-1/PD-L1–directed peptides (therapeutic & imaging), with preclinical/clinical evidence and first mention.

| Target | Name | Development codes / synonyms | Mechanism | Status / Clinical trace | Sponsor | First mention (year) | Reference |
| --- | --- | --- | --- | --- | --- | --- | --- |
| PD-1/PD-L1 | AUNP-12 | AUNP-12 | Peptidic antagonist (PD-1 axis) | Historical (reports of early clinical exploration) | Aurigene | 2013 | (42) |
| PD-L1 | WL12 family | WL12; ^68Ga-WL12; NOTA-WL12 | High-affinity PD-L1-binding peptide (imaging ligand) | First-in-human PET imaging published | Academic / consortium | 2021 | (43) |
| PD-L1 | IMMH-010 / YPD-29B (prodrug/active) | YPD-29B; YPD-30 | Oral peptidomimetic features; PD-L1 binder/dimerizer | NCT / announced; mechanistic paper | CAMS & partners | 2022 | (40) |

Table S1D. Heterocyclic small molecules targeting PD-1/PD-L1 (clinical + tool chemotypes).

| Target | Chemotype / Name | Development codes / synonyms | Format / Mechanism | Status | Sponsor | First mention (year) | Reference |
| --- | --- | --- | --- | --- | --- | --- | --- |
| PD-L1 | Evixapodlin | GS-4224 | Oral PD-L1 binder/dimerizer | NCT04049617; JITC 2024 biomarker paper | Gilead | 2024 | (37) |
| PD-L1 | INCB099280 | INCB099280 | Oral PD-L1 binder/dimerizer | ASCO 2024 #2608 poster | Incyte | 2024 | (39) |
| PD-L1 | INCB086550 | INCB086550 | Oral PD-L1 binder/dimerizer | Translational publication | Incyte | 2022 | (38) |
| PD-L1 | MAX-10181 | MAX-10181 | Oral PD-L1 binder/dimerizer | NCT04122339 | MaxiNovel | 2019 | NCT04122339 |
| PD-L1 | CCX559 | CCX559 | Oral PD-L1 binder | Company / PLoS One pharmacology | ChemoCentryx / Amgen | 2023 | (36) |
| PD-L1 | ABSK043 | ABSK043 | Oral PD-L1 binder | Ann Oncol 2023 abstract; ESMO Asia update | Abbisko | 2023 | (34) |
| PD-L1 | AN4005 | AN4005 | Oral PD-L1 binder | NCT04999384 | Adlai Nortye | 2024 | NCT04999384 |
| PD-L1 | BMS-202 class | BMS-202; PCC0208025 | Biphenyl/heteroaryl PD-L1 dimerizers (tool chemotype) | Benchmark co-crystal and in vitro | BMS | 2016 | (44) |
| PD-L1 | BMS-103 / BMS-142 | BMS-103; BMS-142 | PD-L1 dimerizers (tool chemotype) | Sci Rep 2019 panel | BMS | 2019 | (45) |
| PD-L1 | Biphenyl C2 series | — | PD-L1 dimerizers (series) | ACS Med Chem Lett 2024 | — | 2024 | (46) |

Table S1E. Nanobody/single-domain antibody formats against PD-L1 (therapeutic & imaging).

| Target | INN / Name | Development codes / synonyms | Format / Mechanism | Status | Sponsor | First mention (year) | Reference |
| --- | --- | --- | --- | --- | --- | --- | --- |
| PD-L1 | Envafolimab | KN035 (SC) | Camelid single-domain antibody fused to Fc; subcutaneous | MSI-H/dMMR label; ongoing trials | Alphamab / 3DMed / Simcere | 2021 | (30) |
| PD-L1 | NM-01 (99mTc) | NM-01; 99mTc-NM-01 | Single-domain anti-PD-L1 antibody for SPECT imaging | First-in-human SPECT publication | Novimab / academic | 2019 | (47) |

**References**

1.	O'Malley DM, Randall LM, Jackson CG, Coleman RL, Hays JL, Moore KN, et al. RaPiDS (GOG-3028): randomized Phase II study of balstilimab alone or in combination with zalifrelimab in cervical cancer. Future Oncol. 2021;17(26):3433-43.

2.	Italiano A, Cassier PA, Lin CC, Alanko T, Peltola KJ, Gazzah A, et al. First-in-human phase 1 study of budigalimab, an anti-PD-1 inhibitor, in patients with non-small cell lung cancer and head and neck squamous cell carcinoma. Cancer Immunol Immunother. 2022;71(2):417-31.

3.	Lickliter JD, Gan HK, Voskoboynik M, Arulananda S, Gao B, Nagrial A, et al. A First-in-Human Dose Finding Study of Camrelizumab in Patients with Advanced or Metastatic Cancer in Australia. Drug Des Devel Ther. 2020;14:1177-89.

4.	Papadopoulos KP, Johnson ML, Lockhart AC, Moore K, Falchook GS, Formenti SC, et al. First-In-Human Study of Cemiplimab Alone or In Combination with Radiotherapy and/or Low-dose Cyclophosphamide in Patients with Advanced Malignancies. Clin Cancer Res. 2020;26(5):1025-33.

5.	Markham A. Dostarlimab: First Approval. Drugs. 2021;81(10):1213-9.

6.	Shi Y, Wu J, Wang Z, Zhang L, Wang Z, Zhang M, et al. Efficacy and safety of geptanolimab (GB226) for relapsed or refractory peripheral T cell lymphoma: an open-label phase 2 study (Gxplore-002). J Hematol Oncol. 2021;14(1):12.

7.	Naing A, Infante J, Goel S, Burris H, Black C, Marshall S, et al. Anti-PD-1 monoclonal antibody MEDI0680 in a phase I study of patients with advanced solid malignancies. J Immunother Cancer. 2019;7(1):225.

8.	Brahmer JR, Drake CG, Wollner I, Powderly JD, Picus J, Sharfman WH, et al. Phase I study of single-agent anti-programmed death-1 (MDX-1106) in refractory solid tumors: safety, clinical activity, pharmacodynamics, and immunologic correlates. J Clin Oncol. 2010;28(19):3167-75.

9.	Patnaik A, Kang SP, Rasco D, Papadopoulos KP, Elassaiss-Schaap J, Beeram M, et al. Phase I Study of Pembrolizumab (MK-3475; Anti-PD-1 Monoclonal Antibody) in Patients with Advanced Solid Tumors. Clin Cancer Res. 2015;21(19):4286-93.

10.	Song Y, Zhou K, Jin C, Qian Z, Hou M, Fan L, et al. Penpulimab for Relapsed or Refractory Classical Hodgkin Lymphoma: A Multicenter, Single-Arm, Pivotal Phase I/II Trial (AK105-201). Front Oncol. 2022;12:925236.

11.	Westin JR, Chu F, Zhang M, Fayad LE, Kwak LW, Fowler N, et al. Safety and activity of PD1 blockade by pidilizumab in combination with rituximab in patients with relapsed follicular lymphoma: a single group, open-label, phase 2 trial. Lancet Oncol. 2014;15(1):69-77.

12.	Demidov L, Kharkevich G, Petenko N, Moiseenko V, Protsenko S, Semiglazova T, et al. A phase III study to access the safety and efficacy of prolgolimab 250 mg fixed dose administered every 3 weeks versus prolgolimab 1 mg/kg every 2 weeks in patients with metastatic melanoma (FLAT). Front Oncol. 2024;14:1385685.

13.	Liu R, Li W, Meng Y, Gao S, Zhang J, Hu X. Phase I study of pucotenlimab (HX008), an anti-PD-1 antibody, for patients with advanced solid tumors. Ther Adv Med Oncol. 2021;13:17588359211020528.

14.	Lakhani N, Cosman R, Banerji U, Rasco D, Tomaszewska-Kiecana M, Garralda E, et al. A first-in-human phase I study of the PD-1 inhibitor, retifanlimab (INCMGA00012), in patients with advanced solid tumors (POD1UM-101). ESMO Open. 2024;9(4):102254.

15.	Lee A. Serplulimab: First Approval. Drugs. 2022;82(10):1137-41.

16.	Hoy SM. Sintilimab: First Global Approval. Drugs. 2019;79(3):341-6.

17.	Naing A, Gainor JF, Gelderblom H, Forde PM, Butler MO, Lin CC, et al. A first-in-human phase 1 dose escalation study of spartalizumab (PDR001), an anti-PD-1 antibody, in patients with advanced solid tumors. J Immunother Cancer. 2020;8(1).

18.	Day D, Park JJ, Coward J, Markman B, Lemech C, Kuo JC, et al. A first-in-human phase 1 study of nofazinlimab, an anti-PD-1 antibody, in advanced solid tumors and in combination with regorafenib in metastatic colorectal cancer. Br J Cancer. 2023;129(10):1608-18.

19.	Yu T, Liu X, Wu CY, Tang Z, Wang H, Schnell P, et al. Clinical dose rationale of tislelizumab in patients with solid or hematological advanced tumors. Clin Transl Sci. 2024;17(3):e13769.

20.	Wei XL, Ren C, Wang FH, Zhang Y, Zhao HY, Zou BY, et al. A phase I study of toripalimab, an anti-PD-1 antibody, in patients with refractory malignant solid tumors. Cancer Commun (Lond). 2020;40(8):345-54.

21.	Markham A. Zimberelimab: First Approval. Drugs. 2021;81(17):2063-8.

22.	Wang J, Zhou C, Yao W, Wang Q, Min X, Chen G, et al. Adebrelimab or placebo plus carboplatin and etoposide as first-line treatment for extensive-stage small-cell lung cancer (CAPSTONE-1): a multicentre, randomised, double-blind, placebo-controlled, phase 3 trial. Lancet Oncol. 2022;23(6):739-47.

23.	McDermott DF, Sosman JA, Sznol M, Massard C, Gordon MS, Hamid O, et al. Atezolizumab, an Anti-Programmed Death-Ligand 1 Antibody, in Metastatic Renal Cell Carcinoma: Long-Term Safety, Clinical Activity, and Immune Correlates From a Phase Ia Study. J Clin Oncol. 2016;34(8):833-42.

24.	Boyerinas B, Jochems C, Fantini M, Heery CR, Gulley JL, Tsang KY, et al. Antibody-Dependent Cellular Cytotoxicity Activity of a Novel Anti-PD-L1 Antibody Avelumab (MSB0010718C) on Human Tumor Cells. Cancer Immunol Res. 2015;3(10):1148-57.

25.	Desai J, Fong P, Moreno V, Frentzas S, Meniawy T, Markman B, et al. A Phase 1/2 study of the PD-L1 inhibitor, BGB-A333, alone and in combination with the PD-1 inhibitor, tislelizumab, in patients with advanced solid tumours. Br J Cancer. 2023;128(8):1418-28.

26.	Brahmer JR, Tykodi SS, Chow LQ, Hwu WJ, Topalian SL, Hwu P, et al. Safety and activity of anti-PD-L1 antibody in patients with advanced cancer. N Engl J Med. 2012;366(26):2455-65.

27.	Clingan P, Ladwa R, Brungs D, Harris DL, McGrath M, Arnold S, et al. Efficacy and safety of cosibelimab, an anti-PD-L1 antibody, in metastatic cutaneous squamous cell carcinoma. J Immunother Cancer. 2023;11(10).

28.	Naing A, Thistlethwaite F, De Vries EGE, Eskens F, Uboha N, Ott PA, et al. CX-072 (pacmilimab), a Probody ((R)) PD-L1 inhibitor, in advanced or recurrent solid tumors (PROCLAIM-CX-072): an open-label dose-finding and first-in-human study. J Immunother Cancer. 2021;9(7).

29.	Massard C, Gordon MS, Sharma S, Rafii S, Wainberg ZA, Luke J, et al. Safety and Efficacy of Durvalumab (MEDI4736), an Anti-Programmed Cell Death Ligand-1 Immune Checkpoint Inhibitor, in Patients With Advanced Urothelial Bladder Cancer. J Clin Oncol. 2016;34(26):3119-25.

30.	Papadopoulos KP, Harb W, Peer CJ, Hua Q, Xu S, Lu H, et al. First-in-Human Phase I Study of Envafolimab, a Novel Subcutaneous Single-Domain Anti-PD-L1 Antibody, in Patients with Advanced Solid Tumors. Oncologist. 2021;26(9):e1514-e25.

31.	Patnaik A, Yap TA, Chung HC, de Miguel MJ, Bang YJ, Lin CC, et al. Safety and Clinical Activity of a New Anti-PD-L1 Antibody as Monotherapy or Combined with Targeted Therapy in Advanced Solid Tumors: The PACT Phase Ia/Ib Trial. Clin Cancer Res. 2021;27(5):1267-77.

32.	Gong J, Cao J, Zhang Q, Xu N, Zhao Y, Xing B, et al. Safety, antitumor activity and biomarkers of sugemalimab in Chinese patients with advanced solid tumors or lymphomas: results from the first-in-human phase 1 trial. Cancer Immunol Immunother. 2022;71(8):1897-908.

33.	Xue J, Xue L, Tang W, Ge X, Zhao W, Li Q, et al. TQB2450 in patients with advanced malignant tumors: results from a phase I dose-escalation and expansion study. Ther Adv Med Oncol. 2024;16:17588359231220516.

34.	Cooper A. 700P First-in-human dose-escalating study of ABSK043, a novel and oral small-molecule inhibitor of PD-L1, in patients with advanced solid tumors. 2023.

35.	Zhang Y, Lu J, Levitz D, Dong X, Spira A, Gutierrez M, et al. 691 A phase I study of AN4005, an orally available PD-L1 inhibitor, in patients with advanced tumors: safety and preliminary efficacy. 2024. p. A792-A3.

36.	Sullivan KMC, Vilalta M, Ertl LS, Wang Y, Dunlap C, Ebsworth K, et al. CCX559 is a potent, orally-administered small molecule PD-L1 inhibitor that induces anti-tumor immunity. PLoS One. 2023;18(6):e0286724.

37.	Odegard JM, Othman AA, Lin KW, Wang AY, Nazareno J, Yoon OK, et al. Oral PD-L1 inhibitor GS-4224 selectively engages PD-L1 high cells and elicits pharmacodynamic responses in patients with advanced solid tumors. J Immunother Cancer. 2024;12(4).

38.	Koblish HK, Wu L, Wang LS, Liu PCC, Wynn R, Rios-Doria J, et al. Characterization of INCB086550: A Potent and Novel Small-Molecule PD-L1 Inhibitor. Cancer Discov. 2022;12(6):1482-99.

39.	Prenen H, Daniel J, Ebiana V. A phase 1 study of the small-molecule PD-L1 inhibitor INCB099280 in select advanced solid tumors: updated safety, efficacy, and pharmacokinetics (PK) results. 2024. p. 2608.

40.	Lai F, Ji M, Huang L, Wang Y, Xue N, Du T, et al. YPD-30, a prodrug of YPD-29B, is an oral small-molecule inhibitor targeting PD-L1 for the treatment of human cancer. Acta Pharm Sin B. 2022;12(6):2845-58.

41.	Lee JJ, Powderly JD, Patel MR, Brody J, Hamilton EP, Infante JR. Phase 1 trial of CA-170, a novel oral small molecule dual inhibitor of immune checkpoints PD-1 and VISTA, in patients with advanced solid tumors or lymphomas. 2017. p. TPS3099.

42.	Sasikumar PG, Ramachandra RK, Adurthi S, Dhudashiya AA, Vadlamani S, Vemula K, et al. A Rationally Designed Peptide Antagonist of the PD-1 Signaling Pathway as an Immunomodulatory Agent for Cancer Therapy. Mol Cancer Ther. 2019;18(6):1081-91.

43.	Zhou X, Jiang J, Yang X, Liu T, Ding J, Nimmagadda S, et al. First-in-Humans Evaluation of a PD-L1-Binding Peptide PET Radiotracer in Non-Small Cell Lung Cancer Patients. J Nucl Med. 2022;63(4):536-42.

44.	Zak KM, Grudnik P, Guzik K, Zieba BJ, Musielak B, Domling A, et al. Structural basis for small molecule targeting of the programmed death ligand 1 (PD-L1). Oncotarget. 2016;7(21):30323-35.

45.	Ganesan A, Ahmed M, Okoye I, Arutyunova E, Babu D, Turnbull WL, et al. Comprehensive in vitro characterization of PD-L1 small molecule inhibitors. Sci Rep. 2019;9(1):12392.

46.	Hec-Galazka A, Tyrcha U, Barczynski J, Bielski P, Mikitiuk M, Gudz GP, et al. Nonsymmetrically Substituted 1,1'-Biphenyl-Based Small Molecule Inhibitors of the PD-1/PD-L1 Interaction. ACS Med Chem Lett. 2024;15(6):828-36.

47.	Xing Y, Chand G, Liu C, Cook GJR, O'Doherty J, Zhao L, et al. Early Phase I Study of a 99mTc-Labeled Anti-Programmed Death Ligand-1 (PD-L1) Single-Domain Antibody in SPECT/CT Assessment of PD-L1 Expression in Non-Small Cell Lung Cancer. Journal of Nuclear Medicine. 2019;60(9):1213-20.
