# Supplementary material for: Late-phase strategies to overcome limitations of PD-1/PD-L1 therapy: a clinical development trajectory through 2030
Source: Front Oncol. 2026 Jul 20;16:1875861. doi: 10.3389/fonc.2026.1875861 (PMC13454932; doi:10.3389/fonc.2026.1875861)
Supplement: Supplementary file 2 [file Table2.docx]

**Table S2A.** Verified late-phase programs targeting immune exclusion and angiogenesis-driven vascular-stromal barriers, 2026–2030

| **No.** | **Program** | **Molecule** | **Phase** | **Study completion date** |
| --- | --- | --- | --- | --- |
| **Strict filter** | | | | |
| 1 | AK112-301 / HARMONi-A (NCT05184712) | ivonescimab + pemetrexed/carboplatin | III | 09.2026 (estimated) (ClinicalTrials) |
| 2 | HARMONi-3 (NCT05899608) | ivonescimab + chemotherapy | III | 31.12.2029 (estimated) (ClinicalTrials) |
| 3 | HARMONi-6 (NCT05840016) | ivonescimab + chemotherapy | III | 31.12.2026 (estimated) (ICHGCP) |
| 4 | ROSETTA-Lung-01 (NCT06712355) | pumitamig / BNT327 + chemotherapy | III | 03.2029 (estimated) (ClinicalTrials) |
| 5 | NCT06616532 | PM8002 + paclitaxel | III | 25.12.2028 (estimated) (ClinicalTrials) |
| 6 | LEAP-012 (NCT04246177) | pembrolizumab + lenvatinib + TACE | III | 25.03.2026 (estimated) (ClinicalTrials) |
| 7 | LEAP-014 (NCT04949256) | pembrolizumab + lenvatinib + chemotherapy | III | 12.06.2026 (estimated) (Merck Clinical Trials) |
| 8 | LEAP-015 (NCT04662710) | pembrolizumab + lenvatinib + chemotherapy | III | 31.03.2026 (estimated) (CenterWatch) |
| 9 | HARMONi (NCT06396065) | ivonescimab + pemetrexed/carboplatin | III | 31.12.2026 (estimated) (ClinicalTrials) |
| 10 | HARMONi-7 (NCT06767514) | ivonescimab | III | 06.2029 (estimated) (ClinicalTrials) |
| 11 | HARMONi-GI2 (NCT06953999) | ivonescimab + chemotherapy ± AK117 | III | 14.05.2028 (estimated) (ClinicalTrials) |
| 12 | HARMONi-GI3 (NCT07228832) | ivonescimab + FOLFOX / bevacizumab + FOLFOX | III | 30.11.2029 (estimated) (ClinicalTrials) |
| 13 | ILLUMINE / GORTEC 2024-04 (NCT07264075) | ivonescimab ± ligufalimab | III | 05.2030 (estimated) (ClinicalTrials) |
| 14 | TALENTACE (NCT04712643) | atezolizumab + bevacizumab + TACE | III | 01.02.2029 (estimated) (ClinicalTrials) |
| **Additional borderline programs** | | | | |
| 15 | KEYNOTE-B96 / ENGOT-ov65 (NCT05116189) | pembrolizumab + paclitaxel ± bevacizumab | III | 25.10.2028 (estimated) (ClinicalTrials) |
| 16 | DUO-O (NCT03737643) | durvalumab + chemotherapy + bevacizumab ± olaparib | III | 30.03.2028 (estimated) (ClinicalTrials) |
| 17 | IMbrave152 / SKYSCRAPER-14 (NCT05904886) | atezolizumab + bevacizumab + tiragolumab | III | 01.09.2026 (estimated) (ClinicalTrials) |

Note. The strict pool includes programs in which the anti-VEGF/VEGFR component or a PD-(L)1×VEGF bispecific construct constitutes the direct conceptual core of the strategy for overcoming vascular-stromal barriers and the immune-excluded phenotype. The additional borderline group includes regimens in which an antiangiogenic component is present but does not represent the sole conceptual backbone of the combination. The reference date used was full study completion rather than the date of the primary analysis alone. In ClinicalTrials.gov, the study completion date corresponds to the time point at which collection of all protocol-defined data has been completed for the last participant.

Abbreviations: TACE, transarterial chemoembolization; VEGF, vascular endothelial growth factor; VEGFR, vascular endothelial growth factor receptor.
