# Supplementary material for: Late-phase strategies to overcome limitations of PD-1/PD-L1 therapy: a clinical development trajectory through 2030
Source: Front Oncol. 2026 Jul 20;16:1875861. doi: 10.3389/fonc.2026.1875861 (PMC13454932; doi:10.3389/fonc.2026.1875861)
Supplement: Supplementary file 3 [file Table3.docx]

**Table S2B.** Verified late-phase programs targeting tumor-cell signaling pathways, 2026–2030

| **No.** | **Program (ID)** | **Molecule / class** | **Phase** | **Study completion date** |
| --- | --- | --- | --- | --- |
| 1 | KRYSTAL-7 (NCT04613596) | adagrasib + pembrolizumab | II/III | 31 Oct 2029 (estimated; study completion) (ClinicalTrials.gov) |
| 2 | Krascendo 2 (NCT06793215) | divarasib + pembrolizumab | III | 31 Oct 2030 (estimated; study completion) (ClinicalTrials.gov) |
| 3 | LITESPARK-022 (NCT05239728) | belzutifan + pembrolizumab | III | 28 Sep 2029 (estimated; study completion) (ClinicalTrials.gov) |
| 4 | LUCERNA (NCT06901531) | zolbetuximab + pembrolizumab + chemotherapy | III | 30 Sep 2028 (estimated; study completion) (ClinicalTrials.gov) |
| 5 | OrigAMI-5 (NCT07276399) | amivantamab + carboplatin + pembrolizumab | III | 18 Jun 2029 (trial end date) (J&J Clinical Trials) |
| 6 | RUBY Part 2 (NCT03981796) | dostarlimab + carboplatin/paclitaxel → maintenance dostarlimab + niraparib (PARP/DDR) | III | 26 Nov 2026 (estimated; study completion) (ClinicalTrials.gov) |
| 7 | KRYSTAL-4 (NCT06875310) | adagrasib + pembrolizumab + platinum-based chemotherapy | III | 07 Sep 2029 (estimated; primary completion); 30 Apr 2032 (estimated; study completion) (ClinicalTrials.gov) |
| 8 | SUNRAY-01 (NCT06119581) | olomorasib + pembrolizumab with or without chemotherapy | III | Nov 2027 (estimated; primary completion); Jan 2031 (estimated; study completion) (ClinicalTrials.gov) |
| 9 | SUNRAY-02 (NCT06890598) | olomorasib + pembrolizumab | III | May 2029 (estimated; primary completion); Feb 2032 (estimated; study completion) (ClinicalTrials.gov) |
| 10 | KANDLELIT-007 (NCT07190248) | calderasib (MK-1084) + SC pembrolizumab | III | 07 Dec 2029 (estimated; primary completion); 06 Aug 2032 (estimated; study completion) (ClinicalTrials.gov) |
| 11 | NCT05933577* | intismeran autogene (V940) + pembrolizumab vs placebo + pembrolizumab | III | 26 Oct 2029 (estimated; primary completion) (ClinicalTrials.gov) |
| 12 | NCT06077760* | intismeran autogene (V940) + pembrolizumab vs placebo + pembrolizumab | III | 25 Jun 2030 (estimated; primary completion) (ClinicalTrials.gov) |

Note. The dates shown correspond to study completion when this date falls within the review window; when only a primary completion date is available within the 2026–2030 interval, this is indicated explicitly.

Abbreviations: DDR, DNA damage response; HIF-2α, hypoxia-inducible factor 2 alpha; PARP, poly(ADP-ribose) polymerase.
