# Supplementary material for: Late-phase strategies to overcome limitations of PD-1/PD-L1 therapy: a clinical development trajectory through 2030
Source: Front Oncol. 2026 Jul 20;16:1875861. doi: 10.3389/fonc.2026.1875861 (PMC13454932; doi:10.3389/fonc.2026.1875861)
Supplement: Supplementary file 4 [file Table4.docx]

**Table S3.** Selected biomarkers supporting interim clinical interpretation of PD-1/PD-L1-based strategies before mature confirmatory endpoints become available

| Marker (assay/method, if applicable) | Readout | Typical use | Example setting | Interpretation value | References |
| --- | --- | --- | --- | --- | --- |
| PD-L1 expression by IHC (22C3) | TPS / CPS | Patient selection | Pembrolizumab; NSCLC, HNSCC, gastric/GEJ | Established predictive marker; cutoff is indication-specific | (1-7) |
| PD-L1 expression by IHC (28-8) | TPS | Complementary PD-L1 assessment | Nivolumab; NSCLC | Similar tumor-cell staining to 22C3 in comparability studies | (8, 9) |
| PD-L1 expression by IHC (SP142) | IC-focused / TPS | Assay-specific use | Atezolizumab; TNBC; urothelial carcinoma | Lower tumor-cell sensitivity than 22C3/28-8/SP263 | (8, 9) |
| PD-L1 expression by IHC (SP263) | TPS | PD-L1 assessment | NSCLC and other solid tumors | Comparable tumor-cell staining to 22C3/28-8 in Blueprint studies | (8, 9) |
| MSI-H/dMMR status | Binary status | Patient selection | Pembrolizumab across MSI-H/dMMR tumors | Established predictive marker of PD-1 sensitivity | (10) |
| Tumor mutational burden (TMB) | Continuous / categorical burden | Context-dependent stratification | Selected PD-1/PD-L1 settings | Putative immunogenicity marker; assay-dependent | (11, 12) |
| ctDNA/MRD kinetics | Serial plasma dynamics | Risk stratification | Perioperative immunotherapy-containing regimens | Emerging marker of residual risk and response depth | (13-15) |

Note. PD-L1 expression and MSI-H/dMMR are the most clinically validated biomarkers in this context, whereas TMB and ctDNA/MRD currently function mainly as context-dependent or emerging tools.

Abbreviations: CPS, combined positive score; ctDNA, circulating tumor DNA; dMMR, deficient mismatch repair; GEJ, gastroesophageal junction; HNSCC, head and neck squamous cell carcinoma; IC, immune-cell score; IHC, immunohistochemistry; MRD, minimal residual disease; MSI-H, microsatellite instability-high; NSCLC, non-small-cell lung cancer; PD-L1, programmed death-ligand 1; TMB, tumor mutational burden; TPS, tumor proportion score; TNBC, triple-negative breast cancer.

**References**

1.	Burtness B, Harrington KJ, Greil R, Soulieres D, Tahara M, de Castro GJ, et al. Pembrolizumab alone or with chemotherapy versus cetuximab with chemotherapy for recurrent or metastatic squamous cell carcinoma of the head and neck (KEYNOTE-048): a randomised, open-label, phase 3 study. Lancet. 2019;394(10212):1915-28.

2.	Fuchs CS, Doi T, Jang RW, Muro K, Satoh T, Machado M, et al. Safety and Efficacy of Pembrolizumab Monotherapy in Patients With Previously Treated Advanced Gastric and Gastroesophageal Junction Cancer: Phase 2 Clinical KEYNOTE-059 Trial. JAMA Oncology. 2018;4(5):e180013.

3.	Harrington KJ, Burtness B, Greil R, Soulieres D, Tahara M, de Castro GJ, et al. Pembrolizumab With or Without Chemotherapy in Recurrent or Metastatic Head and Neck Squamous Cell Carcinoma: Updated Results of the Phase III KEYNOTE-048 Study. Journal of Clinical Oncology. 2023;41(4):790-802.

4.	Kwon M, An M-H, Klempner SJ, Lee H, Kim K-M, Sa J-K, et al. Efficacy of Pembrolizumab Monotherapy for Advanced Gastric/Gastroesophageal Junction Cancer with Programmed Death Ligand 1 Combined Positive Score 10 or Greater. Clinical Cancer Research. 2021;27(7):1923-31.

5.	Reck M, Rodriguez-Abreu D, Robinson AG, Hui R, Csoszi T, Fulop A, et al. Pembrolizumab versus Chemotherapy for PD-L1-Positive Non-Small-Cell Lung Cancer. New England Journal of Medicine. 2016;375(19):1823-33.

6.	Rha SY, Oh D-Y, Yanez P, Bai Y, Ryu M-H, Lee J, et al. Pembrolizumab plus chemotherapy versus placebo plus chemotherapy for HER2-negative advanced gastric cancer (KEYNOTE-859): a multicentre, randomised, double-blind, phase 3 trial. Lancet Oncology. 2023;24(11):1181-95.

7.	Shitara K, Van Cutsem E, Bang Y-J, Fuchs C, Wyrwicz L, Lee K-W, et al. Efficacy and Safety of Pembrolizumab or Pembrolizumab Plus Chemotherapy vs Chemotherapy Alone for Patients With First-line, Advanced Gastric Cancer: The KEYNOTE-062 Phase 3 Randomized Clinical Trial. JAMA Oncology. 2020;6(10):1571-80.

8.	Hirsch FR, McElhinny A, Stanforth D, Ranger-Moore J, Jansson M, Kulangara K, et al. PD-L1 Immunohistochemistry Assays for Lung Cancer: Results from Phase 1 of the Blueprint PD-L1 IHC Assay Comparison Project. Journal of Thoracic Oncology. 2017;12(2):208-22.

9.	Tsao M-S, Kerr KM, Kockx M, Beasley MB, Borczuk AC, Botling J, et al. PD-L1 Immunohistochemistry Comparability Study in Real-Life Clinical Samples: Results of Blueprint Phase 2 Project. Journal of Thoracic Oncology. 2018;13(9):1302-11.

10.	Le DT, Uram JN, Wang H, Bartlett BR, Kemberling H, Eyring AD, et al. PD-1 Blockade in Tumors with Mismatch-Repair Deficiency. N Engl J Med. 2015;372(26):2509-20.

11.	Esposito Abate R, Pasquale R, Sacco A, Simeon V, Maiello MR, Frezzetti D, et al. Harmonization of tumor mutation burden testing with comprehensive genomic profiling assays: an IQN Path initiative. Journal for ImmunoTherapy of Cancer. 2024;12(2):e007800.

12.	Rizvi NA, Hellmann MD, Snyder A, Kvistborg P, Makarov V, Havel JJ, et al. Cancer immunology. Mutational landscape determines sensitivity to PD-1 blockade in non-small cell lung cancer. Science. 2015;348(6230):124-8.

13.	Kelly RJ, Landon BV, Zaidi AH, Singh D, Canzoniero JV, Balan A, et al. Neoadjuvant nivolumab or nivolumab plus LAG-3 inhibitor relatlimab in resectable esophageal/gastroesophageal junction cancer: a phase Ib trial and ctDNA analyses. Nature Medicine. 2024;30(4):1023-34.

14.	Tuaeva NO, Falzone L, Porozov YB, Nosyrev AE, Trukhan VM, Kovatsi L, et al. Translational Application of Circulating DNA in Oncology: Review of the Last Decades Achievements. Cells. 2019;8(10):1251.

15.	Valenza C, Saldanha EF, Gong Y, De Placido P, Gritsch D, Ortiz H, et al. Circulating tumor DNA clearance as a predictive biomarker of pathologic complete response in patients with solid tumors treated with neoadjuvant immune checkpoint inhibitors: a systematic review and meta-analysis. Annals of Oncology. 2025;36(7):726-36.
